# Testing a chemical series inspired by plant stress oxylipin signalling agents for herbicide safening activity

**DOI:** 10.1002/ps.4859

**Published:** 2018-02-06

**Authors:** Melissa Brazier‐Hicks, Kathryn M Knight, Jonathan D Sellars, Patrick G Steel, Robert Edwards

**Affiliations:** ^1^ Agriculture, School of Natural and Environmental Sciences Newcastle University Newcastle ‐Upon‐Tyne UK; ^2^ Croda Crop Care Cowick Hall Goole UK; ^3^ School of Pharmacy Newcastle University Newcastle ‐Upon‐Tyne UK; ^4^ Department of Chemistry University of Durham Durham UK

**Keywords:** chloroacetanilides, fenclorim, glutathione transferase, oxylipin, rice

## Abstract

**BACKGROUND:**

Herbicide safening in cereals is linked to a rapid xenobiotic response (XR), involving the induction of glutathione transferases (GSTs). The XR is also invoked by oxidized fatty acids (oxylipins) released during plant stress, suggesting a link between these signalling agents and safening. To examine this relationship, a series of compounds modelled on the oxylipins 12‐oxophytodienoic acid and phytoprostane 1, varying in lipophilicity and electrophilicity, were synthesized. Compounds were then tested for their ability to invoke the XR in Arabidopsis and protect rice seedlings exposed to the herbicide pretilachlor, as compared with the safener fenclorim.

**RESULTS:**

Of the 21 compounds tested, three invoked the rapid GST induction associated with fenclorim. All compounds possessed two electrophilic carbon centres and a lipophilic group characteristic of both oxylipins and fenclorim. Minor effects observed in protecting rice seedlings from herbicide damage positively correlated with the XR, but did not provide functional safening.

**CONCLUSION:**

The design of safeners based on the characteristics of oxylipins proved successful in deriving compounds that invoke a rapid XR in Arabidopsis but not in providing classical safening in a cereal. The results further support a link between safener and oxylipin signalling, but also highlight species‐dependent differences in the responses to these compounds. © 2018 The Authors. *Pest Management Science* published by John Wiley & Sons Ltd on behalf of Society of Chemical Industry.

## 1 INTRODUCTION

Safeners are a chemically diverse group of agrochemicals that enhance tolerance to both pre‐ and post‐emergence herbicides in cereal crops when applied as seed treatments, or as co‐formulants.^1^, ^2^ In contrast to their activity in cereals, safeners do not deliver enhanced herbicide tolerance in non‐domesticated grasses and broad‐leaf crops and weeds. As such, they are important active components included in the formulation of many herbicides used selectively in post‐emergence applications in wheat, barley, rice, maize and sorghum.^1^, ^2^ While safeners have been used commercially for over 40 years, we know remarkably little with respect to their mode of action, although over the course many studies, it is now widely accepted that their action is linked to enhanced herbicide detoxification in cereals.^3^ However, it is likely that additional cytoprotective mechanisms linked to antioxidant capacity are also invoked by safening.^4^ Safener‐induced changes in the biosystem responsible for detoxification (the xenome)^5^ include changes in the expression of a comprehensive group of cytochrome P450s (CYPs), glucosyltransferases, glutathione transferases (GSTs) and ATP‐Binding Cassette (ABC)‐transporter proteins.^6^, ^7^ Curiously, many xenome components are also induced by safeners in broad‐leaf plants and wild grasses, even though this does not result in protection against herbicide injury.^5^, ^8^, ^9^, ^10^ It therefore appears that safener‐signalling pathways are conserved in higher plants, and this has facilitated the use of Arabidopsis as a model system to study their action *in planta*.^5^, ^7^, ^8^, ^11^

**Figure 1:**
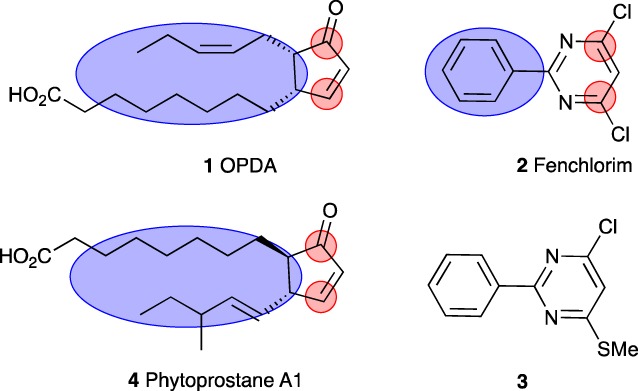
Structures of the oxylipins OPDA **1** and PPA1 **X**, the safener fenclorim **2** and its closely related derivative 3‐chloro‐2‐phenyl‐6‐thiomethiomethylpyrimidine (CMTP) **3**. Unlike fenclorim, CMTP does not invoke a rapid XR or safening and was used as a negative control. For each compound, red circles denote electrophilic centres and the blue regions lipophilic components.

In earlier studies, we determined that the rice safener fenclorim (Fig. 1; compound **2**) provoked a rapid and major induction of xenome genes in Arabidopsis (At), with the tau (U) class GSTs *AtGSTU19* and *AtGSTU24* being particularly responsive.^5^ By studying the induction kinetics of these genes during exposure to a chemical series derived from fenclorim, it was possible to define two classes of xenobiotic response (XR), namely a rapid and a slow XR.^5^ The rapid XR was associated with the induction of GST gene transcription within 20 min of exposure to the test chemical and the transient induction of multiple defence genes as determined using a DNA array.^5^ In contrast, the slow XR only elicited GST gene synthesis 60 min after chemical exposure and resulted in a slower mass perturbation of the transcriptome. While none of the chemical treatments resulted in enhanced herbicide tolerance in Arabidopsis, the fenclorim derivatives provoking the rapid XR, safened rice seedlings against the chloroacetanilide herbicide pretilachlor.^5^ Within the fenclorim chemical series, the characteristics associated with functional safening and the rapid XR were the presence of a pyrimidyl ring containing two electrophilic centres and a suitably orientated and sized hydrophobic substitution (Fig. 1). The chemistry of safener activity was highly defined. Thus, the closely related compound 3‐chloro‐2‐phenyl‐6‐thiomethiomethylpyrimidine (CMTP; Fig. 1; compound **3**), which lacked the electrophilicity of fenclorim, only provoked a slow XR in Arabidopsis and no protection against herbicides in rice.

This well‐defined chemical reactivity and structural features of safening by fenclorim were indicative of selective binding of a safener to a protein target.^12^ In this case of fenclorim, our studies have suggested that this putative protein target must be involved in regulating XR signalling in Arabidopsis, and safening in cereals. However, despite extensive probing for such ligand binding, no protein receptor has been identified.

Instead, we have considered whether the chemical signature of the safening activity of fenclorim could point to orthologous natural signalling molecules that act on the XR pathway. Comparison of microarray experiments in Arabidopsis shows that many of the genes induced by fenclorim^5^ are similarly perturbed following treatment with the oxylipins 12‐oxophytodienoic acid (OPDA) and phytoprostane A1 (PPA1).^13^ Oxylipins are small lipophilic signalling agents released as a result of pathogen infection, wounding and herbivory by insects and as such have a central role in plant stress responses, development and growth.^14^ A significant body of literature has now been established suggesting that safeners and oxylipins share common signalling pathways, although the mechanisms by which these compounds act to enhance xenome transcription remain undefined.^2^, ^13^ In this study, we have postulated that fenclorim directly mimics PPA1‐ and OPDA‐like intermediates, directly acting on the oxylipin signalling pathway. We base this hypothesis on several key chemical similarities between the safener and these cyclopentenones. Whilst there are distinct structural differences between fenclorim and OPDA, the gross molecular shapes have certain similarities, notably the apparent superposition of putative electrophilic centres and hydrophobic residues (Fig. 1). To test this hypothesis, we now report on the preparation and testing for the rapid XR in Arabidopsis and herbicide safening in rice seedlings of a series of hydrophobic and electrophilic compounds inspired by oxylipin chemistry.

## 2 MATERIALS AND METHODS

### 2.1 Preparation and testing of a safener series

Pretilachlor and the commercial safener fenclorim were obtained from Greyhound/Chem Service (Birkenhead, UK, CH41 1LT). *Arabidopsis thaliana* Columbia (Col‐0) cell cultures were maintained as described previously and used 5 days after subculture.^5^ All chemicals were prepared as stock solutions in acetone, such that they were added to the cell cultures as a 1:1000 dilution to achieve final dosing concentrations. Controls consisted of identical volumes of acetone added alone. For herbicide safening trials, rice seedlings (*Oryza sativa ssp. japonica* cv. Nipponbare) were germinated on 0.3% agar containing pretilachlor (10 μM) and safener treatments applied at either 1 or 10 μm, as described previously.^5^

### 2.2 Synthesis of model safeners

**Figure 2:**
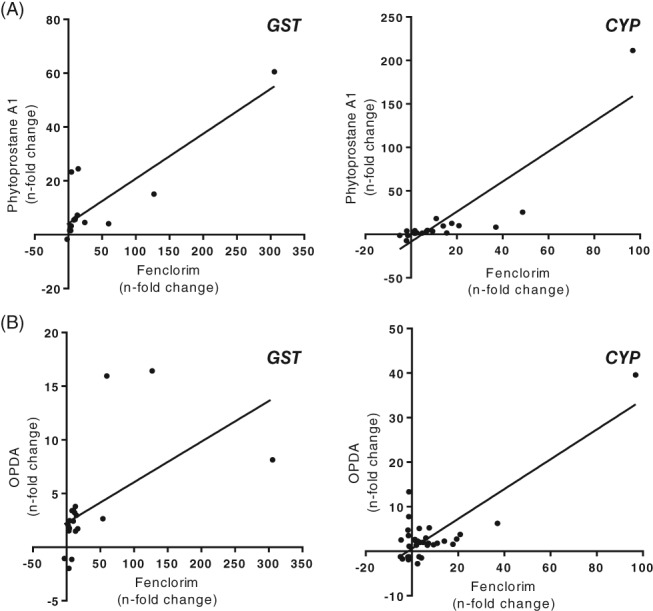
Scatterplots showing the correlation of expression of glutathione transferases (GSTs) and cytochrome P450 (CYP) transcripts after exposure of Arabidopsis cell cultures to the safener fenclorim, or the oxylipin phytoprostane A1 (A) or OPDA (B). The n‐fold change is referenced to a solvent control and is derived from averages of triplicate microarray analysis with a P‐value <0.005.

Compounds numbered in the text in **bold** are shown in Fig 2. Compounds **5–8, 12**, **21** and **22** were obtained commercially from Sigma Aldrich (Gillingham, UK, SP8 4XT) and were used as supplied with no further purification. All other compounds were prepared as described in the Supporting Information.

### 2.3 Real‐time quantitative polymerase chain reaction (qPCR)

Equal amounts of Arabidopsis RNA were used to synthesize cDNA for real‐time PCR of *atgstu19* and *atgstu24* as described previously, with *Glyceraldehyde phosphate dehydrogenase (GAPDH)* (At1g13440) and *Ubiquitin-conjugating (UBC)* (At5g25760) used as housekeeping genes.^5^

### 2.4 Informatics

genevestigator 15 was used to retrieve microarray data for studies with oxylipins.^13^ The three studies used in the analysis were the treatment of Arabidopsis Col‐0 root cultures with 100 μm fenclorim for 4 h (experiment ID: AT‐00459), treatment of Arabidopsis cell cultures with 75 μm PPA1 for 4 h (experiment ID: AT‐00293) and treatment of Arabidopsis Col‐0 plant samples with 75 μm OPDA for 4 h (experiment ID: AT‐00293). Microarray data for all GSTs and CYPs from Arabidopsis (*P*‐value<0.05) was used to construct scatterplots. Regression analysis was performed and Pearson coefficients calculated to determine the correlation between treatment types using the minitab 17 Mintab.com, Coventry, UK. CV3 2TE statistical package.

Phylogenetic analysis was performed as described previously^7^ using the phylogeny package phylip.^16^ clustalw was used to align multiple full‐length protein sequences, the distance measure between paired sequences was computed using prodist, and the UMGMA option in neighor was used to used to construct a tree using an average‐linkage method of clustering. The phylogeny was plotted using drawtree.

## 3 RESULTS

### 3.1 Correlation of xenome gene expression between fenclorim and oxylipin treatments

To focus our studies on the comparative signalling of oxylipins and fenclorim, we compared the relative induction of genes functionally linked to safening rather than performing global transcriptome analyses. Using published DNA array experiments in Arabidopsis, we compared the respective induction of members of the CYP and GST gene superfamilies following PPA1, OPDA and fenclorim treatment, as both groups of enzymes are known to have critical roles in safening in cereals.^1^, ^2^ A strong positive correlation was observed between fenclorim and PPA1 (Fig. 2A) for both the GST and CYP gene families, with Pearson coefficients of 0.852 and 0.882, respectively (both *P*‐values < 0.001). A similar strong correlation was observed between fenclorim and OPDA (Fig. 2B) for the CYP family, but was less pronounced for the GST family, with Pearson coefficients of 0.830 (*P* < 0.001) and 0.558 (*P* = 0.009) respectively. The similarity in PPA1‐ and fenclorim‐invoked changes in GST and CYP gene induction gave further credibility to a mechanistic link between oxylipin and safener signalling in Arabidopsis. The subtle differences seen between OPDA and fenclorim/PPA1 induction of the GST family also indicated an unexpected chemical selectivity in gene regulation.

### 3.2 Preparation of a chemical series inspired by oxylipin chemistry

**Figure 3:**
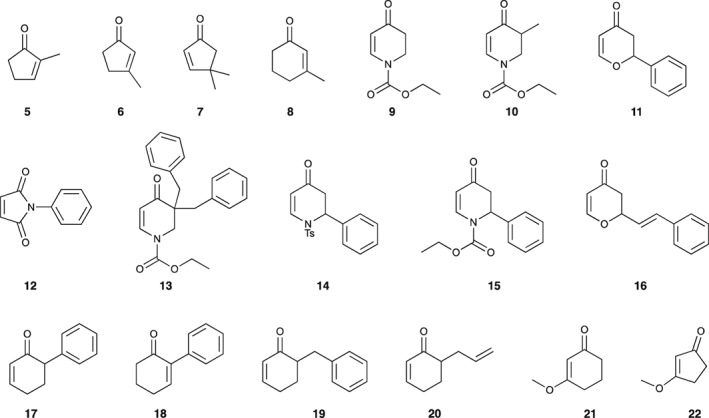
Oxylipin inspired ‘safeners’ explored in this study. Details of their preparation are given in the Supporting Information.

As indicated above, simple structural analysis revealed that both the enone functionality in oxylipins and the dichloropyrimidine moiety within fenclorim are potential 1,3‐bis electrophiles with a hydrophobic element that, we speculated, serves to orientate this warhead to interact with its cognate protein partner. Following these very simple design principles, a set of easily accessible structures were assembled to explore the need for these features whilst retaining elements of similar reactivity (Fig. 3). Compounds **5**–**7** are direct oxylipin analogues with a reactive cyclopentenone unit, albeit lacking the directing lipophilic tail. *N‐*Phenyl maleimide (**12**) is an analogue of known protein modification reagents demonstrating enhanced electrophilicity within the five‐membered ring, whereas **22** retains a similar shape, but possesses lower chemical reactivity**.** All other compounds had a more “fenclorim‐like” six membered ring warhead of varying electrophilicity, ranging from direct “oxylipin‐like” enones to the much less reactive dihydropyridones. Within this latter group, compounds were further selected with small differences in the orientation and size of the lipophilic anchor group. In most cases, these anchor groups reflected the structure of fenclorim, notably containing a simple benzene ring. These compounds were either commercially available, or easily prepared in a few simple steps. In brief, dihydropyridones (**14** and **15**) and pyrones (**11** and **16**) were accessed through heteroatom Diels–Alder reactions using Danishefsky's diene with the relevant imine or aldehyde. Compound **9** was synthesized from 4‐methoxypyridine by sequential reduction, acylation and then acid‐mediated hydrolysis, and acted as a simple precursor of **10** and **13** through enolization and alkylation. Similarly, enones **19** and **20** were generated by alkylation of the corresponding enolate (see Supporting Information for details).

### 3.3 Compound testing in Arabidopsis cultures and rice seedlings

**Figure 4:**
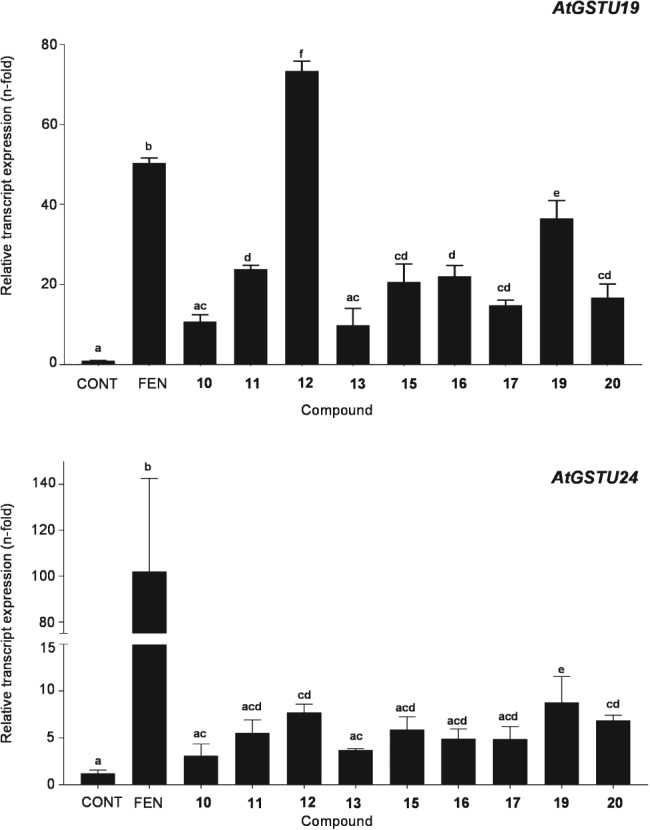
Relative transcript expression of safener‐inducible marker genes in Arabidopsis cell cultures after a 60‐min exposure to the compounds shown in Fig. 3. Each point represented average transcript expression in two independent samples (n = 2; mean ± standard deviation). Different letters represent a statistically significant difference [one‐way ANOVA, Tukey HSD (post hoc); P ≤ 0.05]. CONT, control; FEN, fenclorim.

Each compound was tested in Arabidopsis cell cultures delivered in acetone at a final concentration of 100 μm. Cultures were also treated with fenclorim as this safener is known to cause a rapid XR, with acetone only used as the negative control. Following the addition of the chemical, the cells were extracted after a 1‐h exposure and the change in abundance of transcripts encoding AtGSTU19 and AtGSTU24 determined by real‐time qPCR, as compared with housekeeping genes. Previous studies had demonstrated that, while the rapid XR was detected after 1 h, the slow XR did not cause any induction of these GST biomarkers until later time‐points. The results demonstrated that compounds **5** to **9**, **18**, **21** and **22** failed to elicit a significant response from either AtGSTU19 or AtGSTU24 (Fig. S1). It seemed likely that compounds **5 t**o **9** lacked sufficient hydrophobibicity to be active, while **21** and **22** were not electrophilic enough. After removing these compounds from the analysis along with **14** which also failed to elicit a response that was significantly different from the control, the remainder were then re‐analysed for GST gene induction in Arabidopsis (Fig. 4). Compounds **11**, **12**, **15**, **16**, **17**, **19** and **20** all resulted in a statistically relevant up‐regulation of AtGSTU19 as compared with control samples (Fig. 4). In the case of compound **12**, the enhancement was also significantly greater than that determined with fenclorim. In contrast, AtGSTU24 proved to be less responsive to the safener series than when treated with fenclorim, which was an order of magnitude more effective in inducing this GST. After applying a one‐way analysis of variance [ANOVA; Tukey honest significant difference (HSD) (post hoc); P ≤ 0.05] statistical treatment, compounds **12**, **19** and **20** were all shown to significantly induce AtGSTU24 as compared with the acetone control.

**Figure 5:**
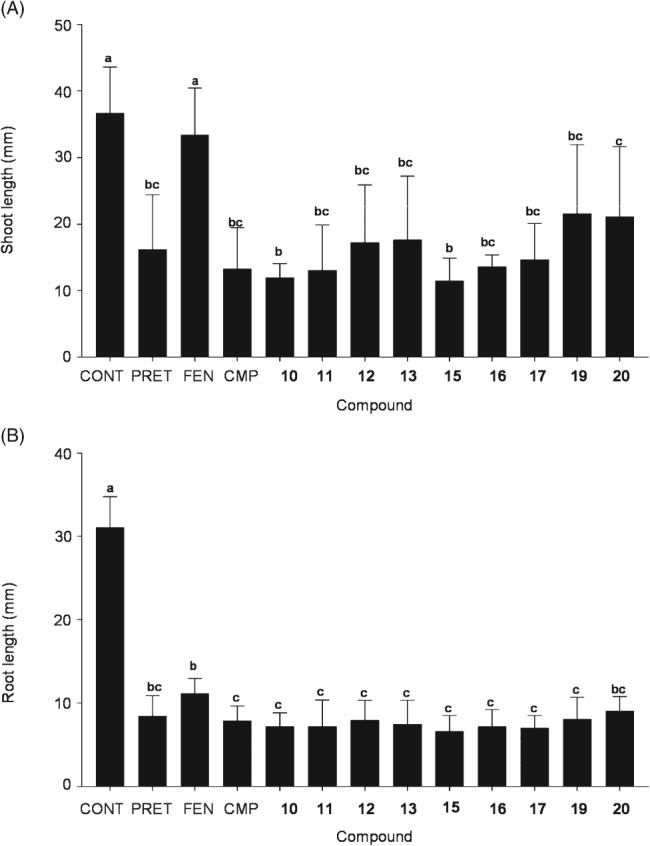
Shoot (A) and root (B) lengths in rice seedlings grown on agar containing 10 μm of the herbicide pretilachlor and 10 μm of the selected safener series compound or 1 μm fenclorim or CMP, as compared with untreated control plants. Each data point represents average length of shoot or root from 15 independent samples (n = 15; mean ± standard deviation). Different letters represent a statistically significant difference [one‐way ANOVA, Tukey HSD (post hoc); P ≤ 0.05]. CONT, no pretilachlor or safener control; PRET, pretilachlor only, no safener compound; FEN, fenclorim **2**; CMP, 3‐chloro‐2‐phenyl‐6‐thiomethiomethylpyrimidine.

For the safening studies with rice, seedlings were germinated and grown on agar containing 10 μm pretilachlor and 10 μm of the test chemical. After 5 days, safening was assessed by determining any protective effect exerted by the chemicals in preventing the stunting of root and shoot growth classically associated with this chloroacetanilide herbicide. As determined from multiple replicates (*n* = 15), considerable variation was observed in both root and shoot lengths receiving the same treatment (Fig. 5). In the roots, no significant safening was determined with fenclorim, or any of the chemical series, with all treatments resulting in reduced growth as compared with untreated controls (Fig. 5B). In the shoots, safening was clearly determined in the presence of fenclorim, which completely reversed the inhibition of shoot growth caused by pretilachlor. On determining mean shoot length, compounds **12**, **13**, **19** and **20** appeared to show a weak protective effect but this was not shown to be significantly different from that of the herbicide pretilachlor on applying one‐way ANOVA analysis (Fig. 5A). However, when the relative induction of the GST biomarkers in Arabidopsis was compared with the effects on shoot growth, a positive correlation was determined (Fig. S2). It could therefore be concluded that, while none of the test series performed as a practical safener, there was a positive correlation between the ability of the compounds to elicit the rapid XR and their ability to provide some protection to the pretilachlor‐treated rice seedlings.

## 4 DISCUSSION

### 4.1 Exploring the relationship between oxylipins and safening

The emerging literature suggests a compelling link between oxylipins and safener signalling, notably in their similar induction of xenome genes.^2,13,14^ In this study, we have directly tested that connection, by designing a group of synthetic compounds inspired by cyclopentenone oxylipins that vary in their electophilicity and hydrophobicity and testing them for their ability to induce the rapid XR in Arabidopsis and safen chloroacetanilide herbicide damage in rice seedlings. The results demonstrated that three compounds from the set, **12**, **19** and **20**, were able to invoke the rapid XR, but did not significantly safen rice seedlings at the concentrations tested. As relative induction of the rapid XR and weak protective effects in rice were positively correlated, it is possible that at higher treatment concentrations these compounds may have provided weak safening. In the case of compounds **12**, **19** and **20**, all three contain the more electrophilic centres (ketone as opposed to analogous vinyl esters and amides), additionally combined with enhanced lipophilicity. Given the lack of any effect shown by **17**, the ability of the maleimide **12,** which is structurally closely related to known nonspecific protein alkylating agents in generating the safening response, suggests that within this series the requirement for electrophilicity is paramount, whereas selectivity is dependent on the lipophilic element.

These observations provide further evidence of a very specific link between the electrophilicity of hydrophobic synthetic and natural product signalling agents and their ability to induce the expression of protective xenome components. Oxylipins form part of a much larger group of stress signalling agents formed from unsaturated fatty acids, collectively termed reactive electrophillic species (RES). Classic RES agents contain hydroperoxide, α,β‐unsaturated epoxides/aldehydes or ketone functions and are distinguished from other fatty acid‐derived signalling agents of altered electrophilicity such as jasmonates bearing saturated ketones/aldehydes and thus are less likely to react with the softer nucleophiles involved in oxylipin processing .^13^, ^14^ Consistent with this, whilst many of these oxidized fatty acids have activity as stress signalling agents, the strong induction of xenome enzymes is restricted to those oxylipins functioning as RES agents.^17^ RES oxylipins induce plant genes bearing activation sequence‐1 (as‐1) cis elements in their promoters, which are in turn regulated by TGA transcription factors , that bind to the regulatory TGACGTCA motif.^18^ Similarly, in animals, there are groups of conserved transcription factors and antioxidant response elements (AREs) controlling the induction of genes by RES.^19^, ^20^ The regulation of ARE‐bearing genes is effected through binding by the leucine zipper transcription factor Nrf2.^19^, ^20^ In turn, the availability of nuclear erythroid 2‐related factor (Nrf2) is controlled by the status of its cognate binding partner, the Kelch‐like ECH‐associated (Keap1) protein. Following the chemical modification of reactive thiol groups of Keap1 by RES agents, Nrf2 is stabilized and released to activate ARE‐bearing genes, thus invoking the xenome response.^18^, ^19^, ^20^ In plants, natural RES agents are known to modify thiol‐bearing proteins, although, as no Keap1 orthologue has yet been identified, the regulatory significance of these interactions is yet to be determined.^14^ However, our studies suggest that any electrophilic modification of a receptor protein is highly specific and determined by specific structural hydrophobic components.

### 4.2 Exploring the links between electophilicity and the activity of commercial safeners

**Figure 6:**
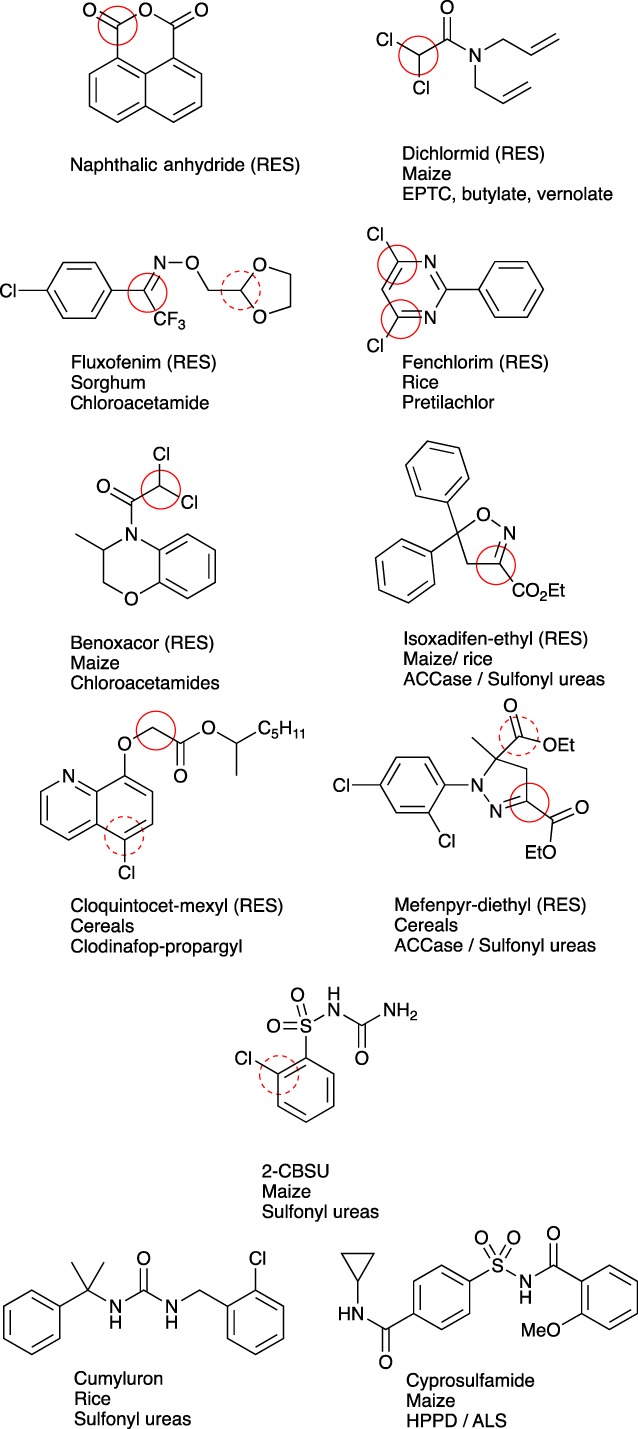
Structureof commercial safeners, with solid red circles denoting electrophilic carbon centres and dashed circles other potential electrophilic groups. Abbreviations: ACCase, acetylCoAcarboxylase; ALS, acetolactate synthase; 2‐CBSU, 4 N‐(aminocarbonyl)‐2‐chlorobenzenesulfonamide) EPTC, S‐ethyl‐N,N‐dipropylthiocarbamate; HPPD, 4‐hydroxyphenylpyruvate dioxygenase).

The correlation between hydrophobicity, electrophilicity and safening was then explored in greater detail. As shown in Fig. 6, many commercial safeners, especially the earlier developed compounds, also bear electrophilic groups and as such qualify as hydrophobic RES agents. In some cases, such as that observed with cloquintocet mexyl and mefenpyr diethyl, the added compound is a pro‐safener. However, the active compounds still retain a balance of electrophilic and hydrophobic residues. Based on recent studies with natural product RES signalling agents in plants and animals,^14,19,20^ it might be anticipated that these electrophilic safeners would invoke the XR and plant protection response to herbicides following the covalent modification of a specific receptor bearing reactive thiol groups, essentially a functional orthologue of the Keap1 protein.^19^, ^20^ Few studies have explored the interactions of safeners with putative receptors and signalling. In the case of dichlormid, binding of the safener to a specific protein in maize has been demonstrated, although this did not involve thiol modification and the identification of the binding partner as a putative phenolic‐O‐methyltransferase has not led to the elucidation of a safening signalling pathway.^12^ Using radiolabelled fenclorim as a probe, we were unable to demonstrate either high‐affinity binding or protein modification in vitro and in planta in either rice or Arabidopsis.^5^, ^11^ From this, we have concluded that either (1) the selective binding and covalent modification of receptors by safeners such as fenclorim involves a very small number of proteins that are below the limit of detection, or (2) the reactivity of fenclorim and its specific interaction with a binding partner are transient and disrupt a dynamic process such as a key catalytic or transport step. If the latter were the case, the obvious pathway for disruption would be the generation and turnover of oxylipins involved in XR signalling.^2^ Whichever mechanism for safener recognition is invoked, it would be anticipated that the signalling pathways they interact with would be conserved in different plant species. However, the chemical diversity of safeners and their crop specificity (Fig. 6) challenge the view that they must act as RES agents on natural stress signalling pathways involving thiol‐bearing receptors. Furthermore, some of the more recent safeners, such as cumyluron and cyprosulfamide, are not overtly electrophilic (Fig. 6). Exploring the relationship between safener structure and activity, we reasoned that if safeners acted on distinct signalling pathways then they should elicit different XRs. The relationship between XR and safener chemistry was then explored using the induction of members of the GST superfamily as a testbed. In the case of Arabidopsis, transcriptome studies have been performed with plants exposed to either fenclorim^5^, ^7^ or a mixture of isoxadifen/mefenpyr‐diethyl. 8 Following the interrogation of the array analyses, we were unable to find a significant correlation between GST expression induced by isoxadifen/mefenpyr‐diethyl treatment and GST expression induced by fenclorim, PPA1 or OPDA treatment (data not shown). This suggests that, at the level of xenome induction, the mixture of isoxadifen/mefenpyr‐diethyl is acting on a fundamentally different branch of the safener signalling pathway from fenclorim, which is more closely associated with the patterns of induction seen with oxylipins.

**Figure 7:**
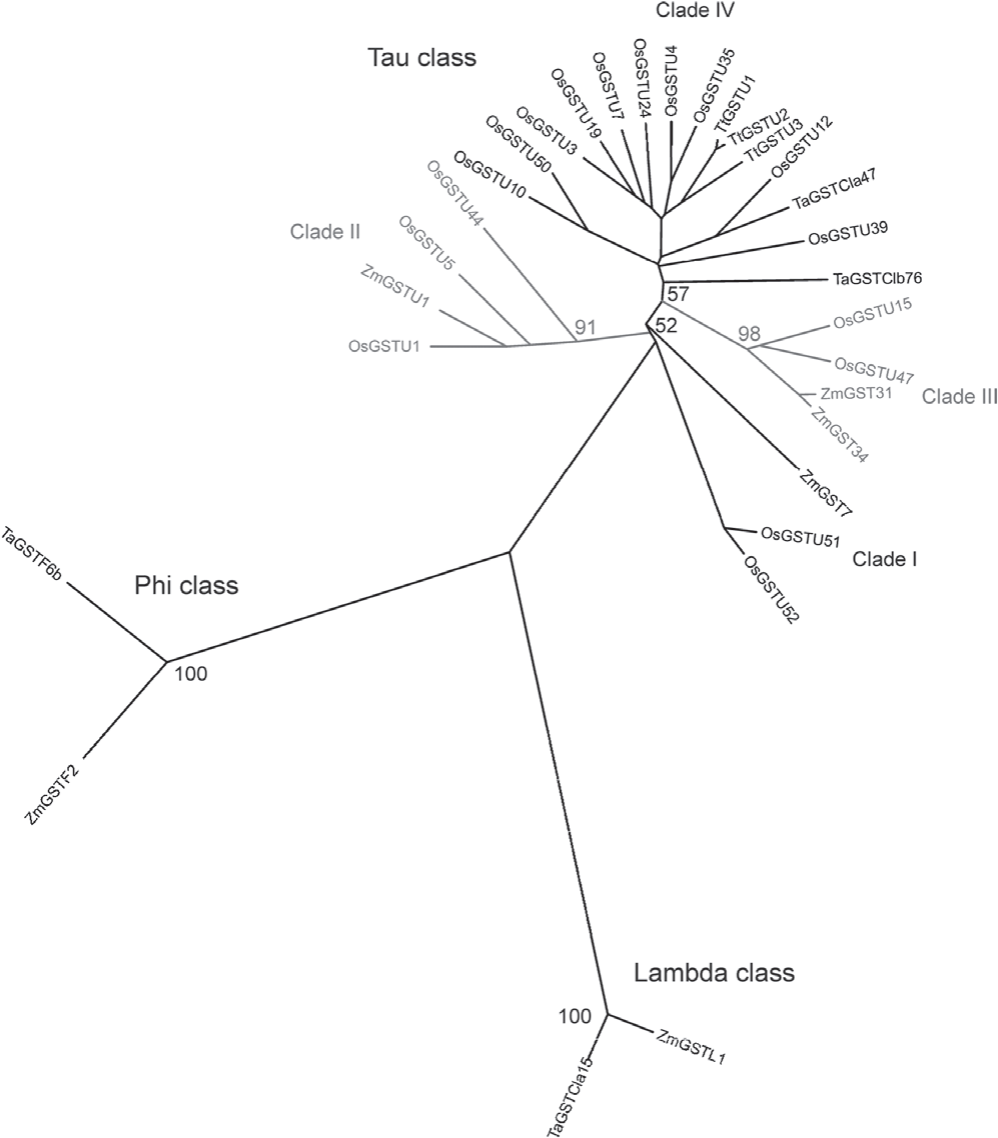
Phylogenetic tree showing the relationship between GSTs from rice (Oryza sativa), maize (Zea mays) and wheat species (Triticum aestivum and Triticum tauschii) induced by herbicide safeners. Rice GSTs (prefix Os) were induced by fenclorim. Maize GSTs (prefix Zm) were induced by dichlormid except for ZmGSTL1, which was induced by N‐(aminocarbonyl)‐2‐chlorobenzenesulfonamide. Cloquinocet mexyl induced GSTs in T. aestivum wheat (prefix Ta) and in the wheat progenitor Triticum tauschii (prefix Tt). Clade I and II tau GSTs are shaded in grey to enhance contrast.

It was then of interest to extend this approach to cereals where the rapid XR is functionally linked to herbicide safening. In a previous study, we had determined the changes in the transcriptome of rice cell cultures treated with 100 μm fenclorim for 4 and 24 h.^7^ From this, it was possible to determine safener‐inducible xenome components and array them in phylogenetic trees to show the relatedness of inducible gene family members (Fig. 7).^7^ A survey of the literature on other transcriptome experiments in cereals revealed a couple of examples of systemic screening of the GST gene family for induction by safeners, namely by subtractive suppression hybridization of wheat treated with cloquintocet mexyl and by microarray analysis of maize exposed to dichlormid.^6^, ^21^ Other safener‐inducible GSTs included in the analysis were identified in gene induction experiments in Triticum tauschii 22 and in maize. 23, 24 Construction of a phylogenetic tree revealed that only a small number of GSTs split between the tau, phi and lambda classes were induced by dichlormid and cloquinocet mexyl in maize and wheat, respectively (Fig. 7). In contrast, a third of rice tau class GSTs were induced by fenclorim, whilst other classes of GSTs were seemingly unperturbed. When taken together with the differences in GST family induction seen with different safeners in Arabidopsis, this does suggest that differing chemistries result in distinctive rapid XRs. This safener specificity in xenome gene induction is particularly intriguing as it may help explain the specificity of these compounds in partnering different herbicides that undergo distinct pathways of detoxification. Safener specificity in xenome induction will now form the basis of future studies in cereals and model species.

## Supporting information


**Appendix S1. MATERIALS AND METHODS**
**Figure S1** Relative transcript expression of safener‐inducible marker genes in Arabidopsis plants 1 h after treatment with different compounds. Each point represented average transcript expression of two independent samples (n = 2, Mean ± SD). Different letter represent statistic difference (one‐way ANOVA, Tukey HSD (posthoc); p ≤ 0.05).
**Figure S2** Scatterplots showing the correlation between biomarker induction in Arabidopsis following treatment with test compounds and the shoot length of rice seedlings grown on agar containing the pretilachlor and test compound.
