# Supplementary material for: Testing a chemical series inspired by plant stress oxylipin signalling agents for herbicide safening activity
Source: Pest Manag Sci. 2018 Feb 6;74(4):828–36. doi: 10.1002/ps.4859 (PMC5873417; doi:10.1002/ps.4859)
Supplement: Supplementary file 1 — Appendix S1. MATERIALS AND METHODS Figure S1 Relative transcript expression of safener‐inducible marker genes in Arabidopsis plants 1 h after treatment with different compounds. Each point represented average transcript expression of two independent samples (n = 2, Mean ± SD). Different letter represent statistic difference (one‐way ANOVA, Tukey HSD (posthoc); p ≤ 0.05). Figure S2 Scatterplots showing the correlation between biomarker induction in Arabidopsis following treatment with test compounds and the shoot length of rice seedlings grown on agar containing the pretilachlor and test compound. [file PS-74-828-s001.docx]

**SUPPORTING INFORMATION FOR**

**Testing a chemical series inspired by plant stress oxylipin signaling agents for herbicide safening activity**

Melissa Brazier-Hicks^a^, Kathryn M. Knight,^b^ Jonathan D. Sellars,^c,d^ Patrick G. Steel^d*^ and Robert Edwards^a^*

^a^ Agriculture, School of Natural and Environmental Sciences, Newcastle University, Newcastle Upon-Tyne, NE1 7RU, UK.

^b^Croda Crop Care,  Cowick Hall, Snaith, Goole, East Yorkshire DN14 9AA

^c^School of Pharmacy, Newcastle University, Newcastle Upon-Tyne, NE1 7RU, UK.

^d^ Department of Chemistry, University of Durham, Durham DH1 3LE, UK.

*Correspondence to: R Edwards, School of Agriculture, Food and Rural Development, Newcastle University, Newcastle Upon-Tyne, NE1 7RU. UK

Email: robert.edwards@ncl.ac.uk. Tel: +44 (0)191 208 6869

&

* P. G. Steel, Department of Chemistry, University of Durham, Durham DH1 3LE, UK

email: [p.g.steel@durham.ac.uk](mailto:p.g.steel@durham.ac.uk). Tel: +44(0)191 334 2131

**MATERIALS AND METHODS**

Compound **5** **- 8**, **12**, **21** and **22** were commercially available from Sigma Aldrich and were used as supplied with no further purification. All other compounds were prepared as described below. All other reagents were obtained from commercial sources and were used as supplied.

**2.2.1. Ethyl 4-oxo-3,4-dihydropyridine-1(2*H*)-carboxylate (9).**  4-Methoxypyridine (2.5 ml, 25 mmol) was dissolved in methanol (50 ml) and treated, in portions, with sodium borohydride (1.0 g, 27 mmol). The resultant mixture was then cooled to -78 °C and ethyl chloroformate (2.6 ml, 27.5 mmol) in ether (5 ml) added dropwise over 30 minutes. The reaction was then stirred at -78 ˚C for a further 1.5 h, treated with H_2_O (30 ml) and warmed to room temperature. The mixture was extracted with EtOAc (3 x 50 ml), dried over MgSO_4_, filtered and concentrated *in vacuo*. Flash chromatography (n-hexane/EtOAc [4:1], [7:3], [3:2]) afforded the title compound as a colourless oil (2.9 g, 69 %); R_f_ 0.2 (n-hexane/EtOAc 9:1); δ_H_ (700 MHz, CDCl_3_) 7.82 (1H, bs, C*H*=CH), 5.31 (1H, bs, CH=C*H*), 4.28 (2H, q, *J* 8, OC*H*_2_CH_3_), 4.00 (2H, t, *J* 8, C*H*_2_), 2.54 (2H, t, *J* 8, C*H*_2_), 1.33 (3H, t, *J* 8, OCH_2_C*H*_3_); δ_C_ (176 MHz, CDCl_3_) 193.4 (*C*=O), 152.0 (*C*=O), 143.4 (*C*H=CH), 107.3 (CH=*C*H), 63.5 (O*C*H_2_CH_3_), 42.4 (*C*H_2_), 35.6 (*C*H_2_), 14.3 (OCH_2_*C*H_3_); *m/z* (ES^+^) 170 (MH^+^); HRMS (ES^+^) Found MH^+^, 170.08086 (C_8_H_12_NO_3_ requires 170.08117). All data agreed with those reported in the literature.^1^

**2.2.2. Ethyl 3-methyl-4-oxo-3,4-dihydropyridine-1(2*H*)-carboxylate (10).** Diisopropylamine (0.10 ml, 0.71 mmol) was dissolved in THF (2 ml), cooled to -78 °C and treated with n-BuLi ([2.5M], 0.28 ml, 0.71 mml). The resultant pale yellow solution was stirred for 15 min, then treated with ethyl 4-oxo-3,4-dihydropyridine-1(2*H*)-carboxylate (0.10 g, 0.60 mmol) in THF (2 ml). The reaction was stirred for another 15 min when methyl iodide (0.06 ml, 0.89 mmol) was added. Subsequently, the reaction was warmed to room temperature over 1.5 h, then mixed with water and extracted with ether (3 x 5 ml). The organic layer was dried over MgSO_4_, filtered, and concentrated *in vacuo*. Flash chromatography (*n*-hexane, *n*-hexane/EtOAc [9:1], [4:1], [7:3], [3:2], [1:1], [2:3], [3:7], [1:4], [1:9], [0:1]) afforded the title compound as a pale yellow solid (23 mg, 21 %); R_f_ 0.7 (*n*-hexane/EtOAc 1:1); δ_H_ (700 MHz, CDCl_3_) 7.80 (1H, bs, C*H*=CH), 5.29 (1H, bs, CH=C*H*), 4.29 (2H, q, *J* 7, OC*H*_2_CH_3_), 4.17 (1H, m, C*H*H), 3.49 (1H, t, *J* 13, CH*H*), 2.56 (1H, septet, *J* 7, C*H*CH_3_), 1.34 (3H, t, *J* 7, OCH_2_C*H*_3_), 1.14 (3H, d, *J* 7, CHC*H*_3_); δ_C_ (176 MHz, CDCl_3_) 195.5 (*C*=O), 152.7 (*C*=O), 142.8 (*C*H=CH), 106.6 (CH=*C*H), 63.5 (O*C*H_2_CH_3_), 48.5 (*C*H_2_), 38.9 (*C*HCH_3_), 14.3 (OCH_2_*C*H_3_), 12.8 (CH*C*H_3_); *m/z* (ES^+^) 184 (MH^+^); HRMS (ES^+^) Found MH^+^, 184.09647 (C_9_H_14_NO_3_ requires 184.09682). All data agreed with those reported in the literature.^2^

**2.2.3. 2-Phenyl-2*H*-pyran-4(3*H*)-one (11).** Benzaldehyde (0.10 ml, 0.94 mmol) was dissolved in toluene (3 ml) and treated with diethylzinc ([1M], 0.09 ml, 0.09 mmol) followed by Danishefsky’s diene (0.37 ml, 1.90 mmol) at room temperature and left to stir for 48 h. The reaction was then quenched with TFA (10 drops), mixed with aq. NaHCO_3_ and extracted with ether (3 x 10 ml). The organic layer was dried over MgSO_4_, filtered, and concentrated *in vacuo*. Flash chromatography (*n*-hexane, *n*-hexane/EtOAc [9:1], [4:1], [7:3], [3:2], [1:1], [2:3], [3:7], [1:4], [1:9], [0:1]) afforded the title compound as a pale yellow oil (76 mg, 46 %); R_f_ 0.6 (n-hexane/EtOAc 3:2); δ_H_ (700 MHz, CDCl_3_) 7.49 – 7.47 (1H, m, C*H*=CH), 7.43 – 7.39 (5H, m, Ar-*H*), 5.53 (1H, d, *J* 6, CH=C*H*), 5.43 (1H, dd, *J* 14, 3, CHC*H*_2_), 2.90 (1H, t, *J* 14, C*H*CH_2_), 2.66 (1H, dd, *J* 14, 3, CHC*H*_2_); δ_C_ (176 MHz, CDCl_3_) 192.4 (*C*=O), 163.4 (*C*H=CH), 138.1 (*ipso*-Ar-*C*), 130.4 (Ar-*C*), 129.2 (Ar-*C*), 126.3 (Ar-*C*), 107.6 (CH=*C*H), 81.3 (*C*HCH_2_), 43.6 (CH*C*H_2_); *m/z* (ES^+^) 175 (MH^+^); HRMS (ES^+^) Found MH^+^, 175.07550 (C_11_H_11_O_2_ requires 175.07536). All data agreed with those reported in the literature.^3^

**2.2.4. Ethyl 3,3-dibenzyl-4-oxo-3,4-dihydropyridine-1(2*H*)-carboxylate (13)** Diisopropylamine (0.3 ml, 2.2 mmol) was dissolved in THF (5 ml), cooled to -78 °C and treated with n-BuLi ([2.5M], 0.9 ml, 2.2 mml). The resultant pale yellow solution was stirred for 15 min then ethyl 4-oxo-3,4-dihydropyridine-1(2*H*)-carboxylate (0.25 g, 1.5 mmol) added in THF (5 ml). The reaction was stirred for a further 15 min and then treated with benzyl bromide (0.34 ml, 3.0 mmol). The reaction was then warmed slowly to room temperature over 1.5 h, and then mixed with water and extracted with ether (3 x 5 ml). The organic layer was dried over MgSO_4_, filtered, and concentrated *in vacuo*. Flash chromatography (*n*-hexane, *n*-hexane/EtOAc [9:1], [4:1], [7:3], [3:2], [1:1], [2:3], [3:7], [1:4], [1:9], [0:1]) afforded the title compound as a colourless oil (15 mg, 3 %); R_f_ 0.6 (*n*-hexane/EtOAc 7:3); υ_max_ (thin film) 3066, 3032, 2994, 2934, 1725 (C=O), 1659, 1600, 1418, 1374, 1296, 1210, 1102, 1008, 898, 752, 701 cm^-1^; δ_H_ (700 MHz, CDCl_3_) 7.62 (1H, bs, C*H*=CH), 7.25 – 7.20 (6H, m, Ar-*H*), 7.10 – 7.09 (4H, m, Ar-*H*), 5.31 (1H, bs, CH=C*H*), 4.29 (2H, m, OC*H*_2_CH_3_), 3.68 (2H, m, C*H*_2_), 3.20 (2H, d, *J* 13, C*H*_2_Ph), 2.56 (2H, d, *J* 13, C*H*_2_Ph), 1.34 (3H, m, OCH_2_C*H*_3_); δ_C_ (176 MHz, CDCl_3_) 196.8 (*C*=O), 152.4 (*C*=O), 142.0 (*C*H=CH), 136.2 (*ipso*-Ar-*C*), 130.6 (Ar-*C*), 128.1 (Ar-*C*), 126.7 (Ar-*C*), 107.4 (CH=*C*H), 63.5 (O*C*H_2_CH_3_), 49.5 (*C*H_2_), 40.6 (*C*H_2_Ph), 14.4 (OCH_2_*C*H_3_); *m/z* (ES^+^) 350 (MH^+^), 372 (MNa^+^); HRMS (ES^+^) Found MH^+^, 350.17519 (C_22_H_24_NO_3_ requires 350.17507).

**2.2.5. *N*-(4’-methylphenylsulfonyl) 2-Pheny-2,3-dihydropyridin-4(1*H*)-one (14) (***E*)-*N*-Benzylidene-4-methylbenzenesulfonamide (0.10 g, 0.4 mmol) was dissolved in toluene (3 ml) and treated with diethylzinc ([1M], 0.04 ml, 0.04 mmol) followed by Danishefsky’s diene (0.11 ml, 0.6 mmol) at room temperature. After stirring at rt for a further 12h the reaction was quenched with aq. NaHCO_3_ and extracted with ether (3 x 10 ml). The organic layer was dried over MgSO_4_, filtered, and concentrated *in vacuo*. Flash chromatography (DCM) afforded the title compound as a pale yellow oil (70 mg, 55 %); R_f_ 0.2 (DCM); δ_H_ (400 MHz, CDCl_3_) 7.80 (1H, dd, *J* 8, 1, C*H*=CH), 7.60 (2H, d, *J* 8, Ar-*H*), 7.23 – 7.14 (7H, m, Ar-*H*), 5.51 (1H, d, *J* 8, CH=C*H*), 5.40 (1H, dd, *J* 8, 1, C*H*CH_2_), 2.83 (1H, dd, *J* 16, 8, CHC*H*_2_), 2.68 (1H, dd, *J* 16, 1, CHC*H*_2_), 2.40 (3H, s, Ar-C*H*_3_); *m/z* (ES^+^) 328 (MH^+^); HRMS (ES^+^) Found MH^+^, 328.10028 (C_18_H_18_NO_3_S requires 328.10019). All data agreed with those reported in the literature.^4,5^

**2.2.6. Ethyl 4-oxo-2-phenyl-1,2,3,4-tetrahydropyridine-1-carboxylate (15).** Ethyl N-[(1*E*)-phenylmethylidene]carbamate^19^ (0.10 g, 0.57 mmol) was dissolved in toluene (3 ml) and treated with diethylzinc ([1M], 0.06 ml, 0.06 mmol), followed by Danishefsky’s diene (0.17 ml, 0.85 mmol) and stirred at room temperature for 36 h. The reaction was then quenched with aq. NaHCO_3_ and extracted with ether (3 x 10 ml). The organic layer was dried over MgSO_4_, filtered, and concentrated *in vacuo*. Flash chromatography (*n*-hexane, *n*-hexane/Et_2_O [9:1], [4:1], [7:3]) afforded the title compound as an off white semi-solid (53 mg, 29 %); δ_H_ (200 MHz, CDCl_3_) 7.51 (1H, d, *J* 12, C*H*=CH), 7.39 – 7.21 (5H, m, Ar-*H*), 5.91 (1H, m, C*H*CH_2_), 5.51 (1H, d, *J* 12, CH=C*H*), 4.1 (2H, m, C*H*_2_), 3.60 (2H, m, C*H*_2_), 1.21 (3H, m, C*H*_3_). All data agreed with those reported in the literature.^6^

**2.2.7. (*E*)-2-Phenyl ethen-2’-yl-2*H*-pyran-4(3*H*)-one (16).** Cinnamaldehyde (0.10 ml, 0.76 mmol) was dissolved in toluene (5 ml) and treated with ytterbium triflate (0.09 g, 0.15 mmol) followed by Danishefsky’s diene (0.18 ml, 0.90 mmol) at room temperature. After stirring at rt for 4 h the reaction was then quenched with aq. NaHCO_3_ and extracted with ether (3 x 10 ml). The organic layer was dried over MgSO_4_, filtered, and concentrated *in vacuo*. Flash chromatography (*n*-hexane, *n*-hexane/EtOAc [9:1], [4:1]) to afforded the title compound as an orange oil (64 mg, 42 %); R_f_ 0.3 (*n*-hexane/EtOAc 9:1); δ_H_ (400 MHz, CDCl_3_) 7.42 – 7.40 (3H, m, Ar-*H*, C*H*=CH), 7.37 – 7.29 (3H, m, Ar-*H*), 6.70 (1H, d, *J* 16, ArC*H*=CH), 6.32 (1H, dd, *J* 16, 6, ArCH=C*H*), 5.47 (1H, d, *J* 6, CH=C*H*), 5.07 (1H, m, C*H*CH_2_), 2.73 (1H, dd, *J* 16, 12, CHC*H*_2_), 2.62 (1H, ddd, *J* 16, 5, 1, CHC*H*_2_); *m/z* (EI) 200 (M+, 45%), 171 (20%), 152 (15%), 128 (100%), 118 (70%), 115 (60%). All data agreed with those reported in the literature.^3^

**2.2.8. 6‐Phenylcyclohex‐2‐en‐1‐one (17) & 2‐Phenylcyclohex‐2‐en‐1‐one (18).**

A solution of 6-bromo-2-phenylcyclohexanone (0.15 g, 0.6 mmol) in 2,6-lutidine (2.5 ml) was heated under reflux for 2 h. The reaction mixture was then concentrated *in-vacuo* and the residue dissolved in Et_2_O (10 ml) and washed with dil. HCl (5 ml) and aq. NaHCO_3_ (5 ml). The organic layer was dried over MgSO_4_, filtered, and concentrated *in vacuo*. Flash chromatography (*n*-hexane/DCM [3:1]) afforded analytical samples of the title compounds:

6‐Phenylcyclohex‐2‐en‐1‐one, **16**: δ_H_ (700 MHz, CDCl_3_) 7.34 – 7.32 (2H, m, Ar-*H*), 7.27 – 7.24 (1H, m, Ar-*H*), 7.16 – 7.15 (2H, m, Ar-*H*), 7.03 (1H, ddd, *J* 4, 3-C*H*), 6.16 (1H, m, 2-C*H*), 3.61 (1H, t, *J* 8, 6-C*H*), 2.49 – 2.46 (2H, m, 4-C*H*_2_), 2.30 – 2.27 (2H, m, 5-C*H*_2_); δ_C_ (176 MHz, CDCl_3_) 199.3 (*C*=O), 150.0 (3-*C*), 139.3 (*ipso*-Ar-*C*), 130.2 (2-*C*), 128.5 (Ar-*C*), 128.2 (Ar-*C*), 126.9 (Ar-*C*), 53.4 (6-*C*), 30.7 (5-*C*H_2_), 25.5 (4-*C*H_2_); *m/z* (EI^+^) 172 (M^+^, 60 %), 104 (100 %).

2‐Phenylcyclohex‐2‐en‐1‐one, **17**: δ_H_ (700 MHz, CDCl_3_) 7.36 – 7.33 (2H, m, Ar-*H*), 7.31 – 7.29 (3H, m, Ar-*H*), 7.03 (1H, t, *J* 4, 2-C=C*H*CH_2_), 2.60 (2H, t, *J* 7, 6-CH_2_), 2.55 (2H, dt, *J* 7, 4, 4-CH_2_), 2.12 (2H, t, *J* 7, 5-C*H*_2_); δ_C_ (176 MHz, CDCl_3_) 197.9 (*C*=O), 147.9 (2-C=*C*H), 140.4 (2-*C*=CH), 136.5 (*ipso*-Ar-*C*), 128.6 (Ar-*C*), 128.0 (Ar-*C*), 127.5 (Ar-*C*), 39.1 (6-*C*H_2_), 26.6 (4-*C*H_2_), 23.0 (5-*C*H_2_); *m/z* (EI^+^) 172 (M^+^, 60 %), 144 (70 %), 130 (65 %), 128 (40 %), 115 (100 %). All data agreed with those reported in the literature.^7^

**2.2.9. 6‐Benzylcyclohex‐2‐en‐1‐one (19).** A solution of diisopropylamine (0.74 ml, 5.3 mmol) in THF (3 ml) was cooled to 0 °C and treated with n-BuLi in hexanes ([1.6M], 3 ml, 4.8 mmol). The solution was stirred for 20 mins at 0 °C , then cooled to -78 °C. 2-Cyclohexen-1-one (0.34 ml, 3.5 mmol) diluted in THF (4 ml) was added dropwise over 10 min and the reaction allowed to stir for 30 min at -78 °C. Benzyl bromide (0.84 ml, 7.0 mmol) was added and stirring continued for a further 30 min before HMPA (2 ml) was added. The reaction was stirred for a further 2 h at -78 °C before being warmed to 0 °C and diluted with Et_2_O (10 ml). The organic layer was washed with sat aq. NH_4_Cl (2 x 10 ml) then brine (2 x 10 ml) and then dried over MgSO_4_, filtered, and concentrated *in vacuo*. Flash chromatography (*n*-hexane, *n*-hexane/EtOAc [9:1], [4:1], [7:3]) to afford the title compound as a colourless oil (0.12 g, 19 %); R_f_ 0.4 (*n*-hexane/EtOAc [9:1]); δ_H_ (700 MHz, CDCl_3_) 7.31 – 7.27 (2H, m, Ar-*H*), 7.23 – 7.17 (3H, m, Ar-*H*), 6.94 (1H, m, 3-C*H*), 6.04 (1H, m, 2-C*H*), 3.37 (1H, dd, *J* 13, 3, 6-C*H*H), 2.58 – 2.48 (2H, m, 6-*H*, 6-CH*H*), 2.40 – 2.34 (1H, m, 4-C*H*H), 2.33 – 2.25 (1H, m, 4-CH*H*), 1.97 (1H, m, 5-C*H*H), 1.65 (1H, m, 5-CH*H*); δ_C_ (176 MHz, CDCl_3_) 200.8 (*C*=O), 149.7 (3-*C*), 139.9 (*ipso*-Ar-*C*), 129.5 (2-*C*), 129.2 (Ar-*C*), 128.4 (Ar-*C*), 126.1 (Ar-*C*), 48.4 (6-*C*), 35.3 (6-*C*H_2_), 27.0 (5-*C*H_2_) 25.3 (4-*C*H_2_); *m/z* (EI^+^) 186 (M+, 60 %), 158 (40 %), 117 (55 %), 95 (100 %). All data agree with those reported in the literature.^8^

**2.2.10. 6‐(Prop‐2‐en‐1‐yl)cyclohex‐2‐en‐1‐one (20).** A solution of diisopropylamine (0.74 ml, 5.3 mmol) in THF (3 ml) was cooled to 0 °C and treated with *n*-BuLi in hexane ([1.6M], 3 ml, 4.8 mmol). The solution was stirred for 20 min at 0 °C, then cooled to -78 °C. 2-Cyclohexen-1-one (0.34 ml, 3.5 mmol) diluted in THF (4 ml) was added dropwise over 10 min and the reaction stirred for 30 min at -78 °C. Allyl bromide (0.61 ml, 7.0 mmol) was then added and eh reaction stirred for a further 30 min when HMPA (2 ml) was added. After a further 2 h at – 78 ˚C, the mixture was warmed to 0 °C and then diluted with Et_2_O (10 ml). The organic layer was extracted with sat aq. NH_4_Cl (2 x 10 ml) and brine (2 x 10 ml) dried over MgSO_4_, filtered, and concentred *in vacuo*. Flash chromatography (*n*-hexane, *n*-hexane/EtOAc [9:1], [4:1], [7:3]) afforded the title compound as a colourless oil (0.12 g, 26%); R_f_ 0.4 (n-hexane/EtOAc [9:1]); δ_H_ (700 MHz, CDCl_3_) 6.93 (1H, m, 3-C*H*), 6.00 (1H, m, 2-C*H*), 5.79 (1H, m, 6-CH_2_C*H*=CH_2_), 5.08 – 4.98 (2H, m, 6-CH_2_CH=C*H*_2_), 2.62 (1H, m, 6-C*H*HCH=CH_2_), 2.40 – 2.32 (3H, m, 6-*H*, 4-C*H*_2_), 2.15 – 2.07 (2H, m, 6-CH*H*CH=CH_2_, 5-C*H*H), 1.72 (1H, m, 5-C*H*H); δ_C_ (176 MHz, CDCl_3_) 200.8 (*C*=O), 149.7 (3-*C*), 136.1 (6-CH_2_*C*H=CH_2_), 129.5 (2-*C*), 116.7 (6-CH_2_CH=*C*H_2_), 46.1 (6-*C*), 33.6 (6-*C*H_2_CH=CH_2_), 27.3 (5-*C*H_2_) 25.2 (4-*C*H_2_); *m/z* (EI^+^) 136 (M^+^, 80 %), 121 (40 %), 107 (45 %), 94 (100 %), 79 (80 %). All data agree with those reported in the literature.^9^

**REFERENCES**

# Sebesta R, Pizzuti MG, Boersma AJ, Minnaard AJ and Feringa BL, Catalytic enantioselective [conjugate addition](javascript:popupOBO('RXNO:0000008','B417727D')) of dialkylzinc reagents to *N*-substituted-2,3-dehydro-4-piperidones. *Chem Commun* 13:1711-1713 (2005).

1. Gradillas A, Belmonte E, d. Silva RF and Pérez‐Castells J, Hydroxy chalcogenide-promoted Morita–Baylis–Hillman alkylation reaction: intermolecular applications with alkyl halides as electrophiles. *Eur J Org Chem* **9**:1935-1941 (2014).
2. Kitazawa T and Mukaiyama T, Hetero Diels-Alder type reactions between Danishefsky's dienes in the presence of Lewis base catalysts. An efficient method for the synthesis of substituted 2,3-dihydropyran-4-ones. *Heterocycles* **69**:417-427 (2006).
3. Zhao GL and Shi M, Aza-Baylis-Hillman reactions of *N*-tosylated aldimines with activated allenes and alkynes in the presence of various Lewis base promoters. *J Org Chem* **70**:9975-9984 DOI (2005).
4. Wenzel AG and Jacobsen EN, Asymmetric catalytic Mannich reactions catalyzed by urea derivatives: enantioselective synthesis of beta-aryl-beta-amino acids. *J Am Chem Soc* **124**:12964-12965 (2002).
5. Dieter RK and Guo F, Conjugate addition reactions of *N*-carbamoyl-4-pyridones with organometallic reagents. *J Org Chem* **74**:3843-3848 (2009).
6. Felpin FX, Practical and efficient Suzuki-Miyaura cross-coupling of 2-iodocycloenones with arylboronic acids catalyzed by recyclable Pd(0)/C. *J Org Chem* **70**:8575-8578 (2005).
7. Magnus P, Lacour J, Evans PA, Rigollier P and Tobler H, Applications of the beta-azidonation reaction to organic synthesis. alpha,beta-enones, conjugate addition, and gamma-lactam annulation. *J Am Chem Soc* **120**:12486-12499 (1998).
8. Pandey G, Adate PA and Puranik VG, Organocatalytic dynamic kinetic resolution via conjugate addition: synthesis of chiral trans-2,5-dialkylcyclohexanones. *Org Biomol Chem* **10**:8260-8267 (2012).

**Figure S1** Relative transcript expression of safener-inducible marker genes in Arabidopsis plants 1h after treatment with different compounds. Each point represented average transcript expression of two independent samples (n = 2, Mean ±SD). Different letter represent statistic difference (one-way ANOVA, Tukey HSD (posthoc); p ≤ 0.05).

**Figure S2** Scatterplots showing the correlation between biomarker induction in Arabidopsis following treatment with test compounds and the shoot length of rice seedlings grown on agar containing the pretilachlor and test compound.
